# Prognostic Role of Circulating DNA in Biliary Tract Cancers: A Systematic Review and Meta-Analysis

**DOI:** 10.3390/cancers17213451

**Published:** 2025-10-28

**Authors:** Sara Boggio, Laura Alaimo, Edoardo Poletto, Alberto Quinzii, Giada Scoccati, Mario De Bellis, Simone Conci, Tommaso Campagnaro, Andrea Ruzzenente

**Affiliations:** 1 General and Hepato-Biliary Surgery Unit, Department of Surgery, Dentistry, Gynecology, and Pediatrics, University of Verona, University Hospital G.B. Rossi, P.le L.A. Scuro 10, 37134 Verona, Italy; 2 Department of Medicine, Division of Oncology, University of Verona, University Hospital G.B. Rossi, 37134 Verona, Italy

**Keywords:** ctDNA, biliary tract cancer, long-term outcomes, chemotherapy, liquid biopsy

## Abstract

**Simple Summary:**

Biliary tract cancer (BTC) is a rare but aggressive malignancy, often diagnosed at advanced stages and associated with limited treatment options and poor prognosis. Current diagnostic approaches, such as tissue biopsy, are invasive and may not adequately capture tumor dynamics or predict treatment response. To address this gap, this systematic review evaluated the prognostic role of circulating tumor DNA (ctDNA) and related biomarkers, particularly variant allele frequency (VAF), in BTC. Across ten high-quality studies involving 2103 patients, ctDNA positivity and elevated VAF levels were consistently associated with worse overall and progression-free survival, regardless of treatment type or sampling time. These findings highlight ctDNA as a promising non-invasive biomarker for risk stratification, relapse detection, and treatment monitoring. By supporting the integration of liquid biopsy into clinical practice, this research advances precision oncology in BTC and lays the groundwork for future studies aimed at refining personalized treatment strategies.

**Abstract:**

Background: Biliary tract cancer (BTC) is an aggressive malignancy often diagnosed at an advanced stage and is associated with a poor prognosis. Non-invasive approaches can facilitate the early detection and identification of biomarkers to inform treatment strategies. Liquid biopsy, particularly through the analysis of circulating tumor DNA (ctDNA), has recently emerged as a valuable clinical and prognostic tool for guiding BTC management. Methods: The PubMed, Cochrane Library, and Wiley databases were searched for terms related to BTC and ctDNA, aiming to include studies evaluating the value of ctDNA as a predictor of overall (OS), progression-free (PFS), disease (DFS), and recurrence-free survival (RFS). Results: Twelve studies encompassing 2374 patients were considered eligible. The detection of ctDNA was associated with higher mortality and progression risk (HR 2.61, 95%CI 2.19–3.11 and HR 2.69, 95%CI 1.82–3.98, respectively), regardless of the ctDNA sampling time. The variant allele frequency (VAF) emerged as a valuable predictive marker, with higher VAF values being associated with higher mortality and progression risk (HR 2.37, 95%CI 1.83–3.06, and HR 2.22, 95%CI 1.40–3.53, respectively) compared with low levels of VAF. This association was observed regardless of chemotherapy administration, suggesting that VAF may serve as a potential marker of treatment resistance. Conclusions: This review underscores the clinical relevance of ctDNA status and related markers, such as VAF, in the management and prognostic evaluation of BTC. The findings support the integration of liquid biopsy into clinical practice to improve risk stratification, enable the early detection of relapse, and inform personalized treatment strategies, ultimately contributing to more precise and effective patient care in BTC.

## 1. Introduction

Biliary tract cancers (BTCs) refer to a variety of malignant tumors, more frequently adenocarcinomas, that originate from the biliary tree (i.e., cholangiocarcinoma [CCA]), gallbladder, and cystic duct (i.e., gallbladder cancer [GBC]) [1]. CCA is classified into two subtypes: extrahepatic (eCCA), which comprises distal (dCCA) and perihilar CCA (pCCA), and intrahepatic CCA (iCCA), which arises proximal to the second-order bile ducts [2,3,4]. Although iCCA is becoming more common in Western countries, partly due to improved disease recognition [5], BTCs remain rare tumors that often present at an advanced stage and carry a poor prognosis [3]. The 5-year survival rate ranges from 20% to 25% for patients diagnosed with resectable disease to less than 5% for those with advanced disease at the time of diagnosis [6].

The current standard of care for high-risk resected BTC is adjuvant capecitabine, either in combination with radiotherapy [7,8]. However, the majority of patients are diagnosed at advanced stages [9], and between 60% and 70% of patients experience recurrence following resection [10,11]. Prognostic assessment and molecular profiling are essential components of patient management following diagnosis, particularly as precision medicine and targeted therapies increasingly guide treatment selection [12]. Early detection and screening are key factors in reducing tumor burden, mortality, and treatment expenses.

The term “liquid biopsy” (LB) encompasses various techniques and detection methods applied to bodily fluids for comprehensive analysis throughout cancer management [13]. This includes molecular profiling, screening for minimal residual disease (MRD), recurrence detection in the adjuvant setting, and treatment selection, thereby allowing a precise approach and enabling the assessment of treatment dynamics [14]. LB offers multiple advantages for early tumor detection compared with solid tissue biopsy including non-invasiveness, a higher turnaround time, and easier sampling and repeated sample collection. LB is used to detect circulating markers such as extracellular vesicles (EVs), tumor-educated platelets (TEPs), circulating tumor cells (CTCs), microRNA (miRNA), circulating tumor DNA (ctDNA), and circulating RNA (ctRNA) [15].

Cells and cellular components originating from primary or metastatic tumor sites are “physiologically” present in the peripheral blood of patients. These include CTCs, circulating free DNA (cfDNA), and exosomes that contain proteins, lipids, and nucleic acids. As tumor cells undergo apoptosis or programmed cell death, they shed DNA fragments [16]. The portion of cfDNA that originates from primary tumors and/or metastases and contains genetic or epigenetic changes, often unique to those tumors, is referred to as ctDNA. In contrast, cfDNA also includes fragments derived from normal/apoptotic and necrotic cells [17]. Both ctDNA and circulating extracellular nucleic acids (i.e., cfDNA) can be isolated from blood samples.

For a variety of cancers including CCA, cfDNA and ctDNA may be used in place of tissue biopsy due to their diagnostic and prognostic value [18] since ctDNA contains somatic mutations of tumor origin. Furthermore, ctDNA has been demonstrated to predict cancer patients’ prognosis and response to treatment [19]. Post-operative monitoring of ctDNA has been shown to have a prognostic role in several cancers, predicting early recurrence after resection [20,21,22]. Additionally, it can track mechanisms of therapy resistance and serve as a marker of treatment response, as demonstrated in colon and breast cancers [23,24]. In the context of hepatobiliary malignancies, ctDNA has been demonstrated to predict early recurrence after surgery for hepatocellular carcinoma (HCC) [25,26].

The analysis of ctDNA can detect unique tumoral genetic changes and enable the serial, precise, and noninvasive monitoring of tumor dynamics as well as treatment selection [27,28,29]. With the increasing adoption of genomic profiling through next-generation sequencing (NGS) techniques in recent years, several studies have described genomic subtypes of BTC characterized by targetable alterations [30,31]. Specifically, up to 55% of BTC patients may harbor ctDNA changes that are considered therapeutically relevant [29] such as FGFR fusions, rearrangements, or IDH1 and IDH2 mutations. Even though LB may be a desirable diagnostic method for early stage BTC, current data are limited, and technical challenges have hindered progress in the field. Additionally, most patients with BTC exhibit low rates of detectable ctDNA and lower ctDNA levels than those with other cancers [32,33].

This systematic review sought to collect and evaluate the potential clinical applications of liquid biopsy in the form of cfDNA/ctDNA as a predictor of prognosis and treatment-related outcomes in BTC.

## 2. Materials and Methods

### 2.1. Search Strategy

A systematic search of the literature was conducted in electronic databases, including PubMed, Wiley, and the Cochrane Library, from database inception to October 2025. The search strategy was tailored to each database to ensure a comprehensive search, using the following terms: “cell”, “free”, “circulating”, “DNA”, “cfDNA”, “ctDNA”, “tumor”, “cancer”, “liver”, “biliary”, “cholangiocarcinoma”, “gallbladder”, “prognosis”, “outcome”. The term “review” was always excluded using the NOT function. All retrieved studies were screened for relevant references. Only studies published in the English language were considered eligible. This review was carried out following the Reporting Items for Systematic Reviews and Meta-analyses (PRISMA) and the Declaration of Helsinki [34]. The study protocol was registered in PROSPERO (ID: 1005111). After the full-text screening, we decided to narrow our attention to BTCs, with the outcome of interest focused on the relationship between ctDNA/cfDNA and prognosis. As such, all the included studies addressed the prognostic role of ctDNA or cfDNA in BTC. CADIMA (version 2.2.4.2, Julius Kühn-Institut, Quedlinburg, Germany) was used to import all the articles that were discovered through the formal search strategy [35]. This tool guarantees a computerized distribution of records throughout the screening process (considering the extent of a possible independent and parallel assessment) and enables automated duplicate removal. Multiple authors screened the studies in parallel (S.B., L.A., E.P., G.S.), and inconsistencies and disagreements were resolved by consensus among authors.

### 2.2. Inclusion and Exclusion Criteria

The studies were considered eligible if they satisfied the following criteria: (1) studies including at least 20 patients with a confirmed diagnosis of BTC; (2) ctDNA or cfDNA was collected from serum, plasma, or peripheral blood; (3) studies reporting ctDNA/cfDNA measurements with available data on overall survival (OS) and/or progression-free survival (PFS). Exclusion criteria included a lack of survival data, reviews, letters, case reports, and abstracts as well as failure to obtain the full text.

### 2.3. Data Extraction and Quality Assessment

Two reviewers (S.B. and L.A.) independently performed the data extraction. Data extracted included study baseline characteristics (i.e., first author, journal, and year of publication, number of patients), patient and tumor characteristics (i.e., age and subtype of BTC cancer), ctDNA-related information (i.e., ctDNA source, collection, detection, and isolation method), and long-term outcomes (i.e., overall [OS], progression-free [PFS] and recurrence-free survival [RFS]). Additionally, data on relapse detection rates were collected, along with the corresponding sensitivity (Sn), specificity (Sp), positive predictive values (PPVs), and negative predictive values (NPVs). When studies did not provide complete data on long-term outcomes, Enguage Digitizer 11.1 software was used to extract the survival rates (http://plotdigitizer.sourceforge.net, accessed on 1 May 2025). The Newcastle–Ottawa Scale (NOS) was used to evaluate the quality of all included studies, and this assessment was performed independently by two researchers (S.B. and L.A.). NOS values were assigned on a scale of 0–8, with scores of 6 or higher indicating high-quality investigations [36].

### 2.4. Statistical Analysis

For descriptive statistics, categorical variables were expressed as counts and percentages (%). The mean (standard deviation [SD]) or median (interquartile range [IQR]) was used to summarize continuous variables. Publication bias was assessed by visual inspection of the funnel plot and formally tested using Egger’s regression test. For the purpose of this analysis, ctDNA was treated as a binary variable (detected vs. not detected). The studies employed the Kaplan–Meier method, log-rank test, and Cox regression analysis to estimate and compare OS, PFS, and RFS between the ctDNA-positive and ctDNA-negative patient subgroups. Both common effects and random effects models were employed to pool hazard ratios (HRs). The inverse variance method was used in the common effects model to calculate the overall HR. On the other hand, the random effects model estimated the between-study variance using the DerSimonian–Laird method, which accounts for heterogeneity. Generally, when I^2^ > 50% or *p*-value < 0.05, heterogeneity is taken into account, and it is recommended to pool HRs using the random effects model, while when I^2^ ≤50%, it is advisable to use the common effects model. Each study’s log (HR) and standard error (SE) log(HR) were used to compute pooled hazard ratios (HRs) with 95% confidence intervals (95%CI). Forest plots were used to illustrate the results. The mathematical characteristics of logarithmic transformations and their impact on values across various scales and ranges were responsible for the slight variations in the transformed HR values reported in the forest plots compared with those reported by the studies. However, the process of transformation and back-transformation maintained the overall pattern and interpretation of the HR ratios [37]. Regression models were built using the R^2^ test, also known as the coefficient of determination. A sensitivity analysis was conducted using a one-by-one elimination method. Review Manager (RevMan, version 5.2 software Metagen package) was used for forest plots. Statistical analyses were conducted utilizing R version 4.2.0 (R Project for Statistical Computing).

## 3. Results

### 3.1. Search Results

**Figure 1:**
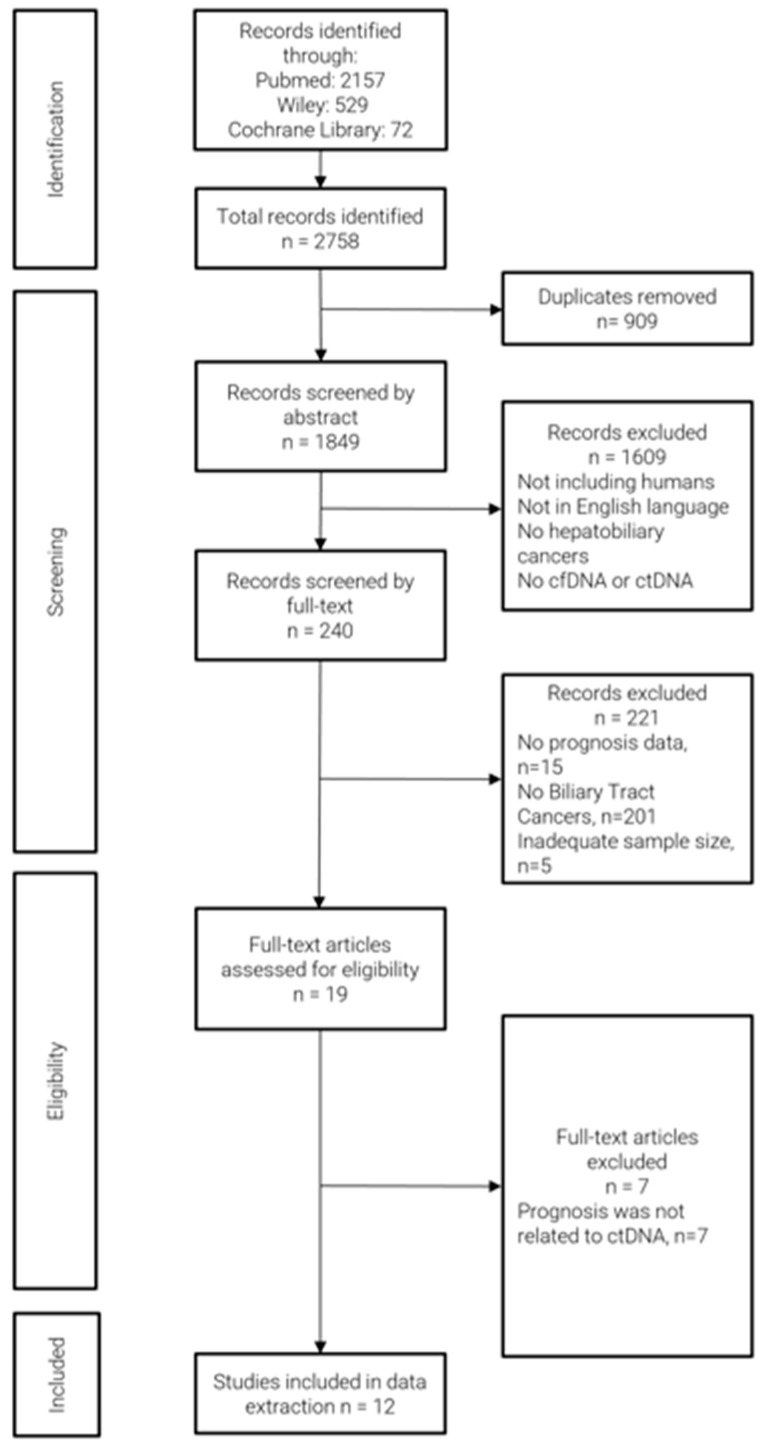
Flow diagram of study selection.

The literature search yielded a total of 2758 articles, which were imported into CADIMA. A total of 19 studies fulfilled the inclusion criteria. However, seven studies were excluded because the prognosis data were not related to ctDNA/cfDNA status or associated markers. As a result, 12 studies were selected, and their data were extracted (Figure 1). All included studies reported prognosis-related data such as OS, PFS, or RFS. The results of the quality assessment of the studies are reported in Supplementary Table S1. Notably, all studies were judged as high-quality investigations with NOS ≥ 6.

A total of 2374 patients out of 2402 with confirmed diagnosis of BTC and a median age of 64.3 years (range 59.0–67.0) were included in the meta-analysis (Table 1). The remaining cases included 19 benign lesions and 9 lesions of unknown origin. Among the total cohort included in this review (*n* = 2374), only 272 (11.46%) patients underwent surgical resection. Of these, 115 (42.3%) patients underwent surgery alone [38,39,40,41], while 143 (52.3%) patients received surgery followed by adjuvant chemotherapy [39,42]. A total of 2235 patients had ctDNA/cfDNA data available. Nine out of twelve studies performed an analysis on ctDNA [38,39,42,43,44,45,46,47,48], while there were three on cfDNA [40,41,49]. In all studies, ctDNA was obtained from plasma. Five studies analyzed treatment-naïve patients [38,39,40,41,49], and eleven analyzed samples from patients receiving systemic therapy [38,39,41,42,43,44,45,46,47,48,49]. The timing of sample collection varied across the studies. Specifically, six studies collected the samples before treatment or at admission for surgery-only cases (treatment-naïve) [38,40,41,44,48,49], whereas two studies analyzed the ctDNA after systemic therapy [46,47], and four studies at multiple time points (before and after treatment) [39,42,43,45] (Table 2). The specific thresholds and parameters used in each study to define ctDNA-positive status were extracted from the original authors’ reports and are summarized in Table 3.

### 3.2. Impact of ctDNA/cfDNA Status on Overall Survival

**Figure 2:**
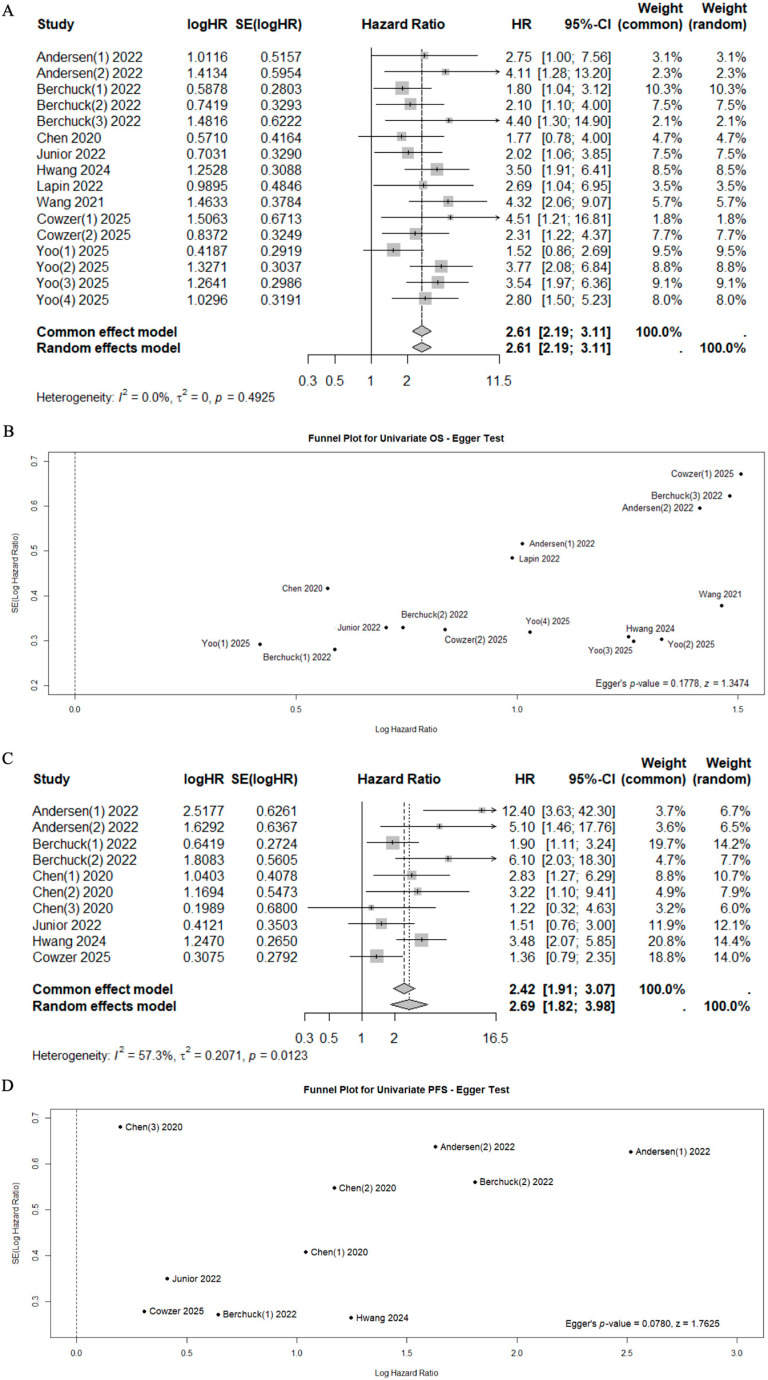
Forest plots for mortality risk (**A**) with funnel plot for publication bias [40,41,42,43,44,46,47,48,49] (**B**), and for progression risk (**C**) with funnel plot for publication bias [41,44,46,47,48,49] (**D**).

A total of 10 studies reported data on OS, PFS, and RFS with univariate or multivariate analyses [39,40,41,42,43,44,46,47,48,49], while 2 studies reported data on relapse rates in relation to ctDNA status (Sn, Sp, PPV, NPV) [38,39] (Table 3). In the univariate analysis, the positivity of ctDNA status was significantly associated with poor outcomes (Mortality HR 2.61, 95%CI 2.19–3.11, I^2^ = 0%, *p* = 0.4925 and Progression HR 2.69, 95%CI 1.82–3.98, I^2^ = 57.3%, *p* = 0.0123, respectively) (Figure 2). Significant heterogeneity was observed for progression risk (I^2^ = 57.3%). However, the sensitivity analysis confirmed the prognostic value of cfDNA/ctDNA status, which remained consistent regardless of specific studies being included or excluded (Supplementary Figure S2).

Among patients with positive ctDNA, median OS and PFS ranged from 2.49 to 38.70 months (median 10.35 months, IQR 8.10–21.25), and from 0.64 to 5.00 months (median 4.20 months, IQR 2.75–4.57), respectively. Conversely, among patients with negative ctDNA, median OS and PFS ranged from 5.20 to 49.70 months (median 20.10 months, IQR 16.18–37.23) and from 2.46 to 16.40 months (median 7.65 months, IQR 4.98–8.28), respectively (Table 4). This pattern was statistically significant for both OS (Mann–Whitney U test, *p* = 0.03) and PFS (Mann–Whitney U test, *p* = 0.03) (Supplementary Figure S1).

### 3.3. Subgroup Analysis on ctDNA Time of Sample Collection

**Figure 3:**
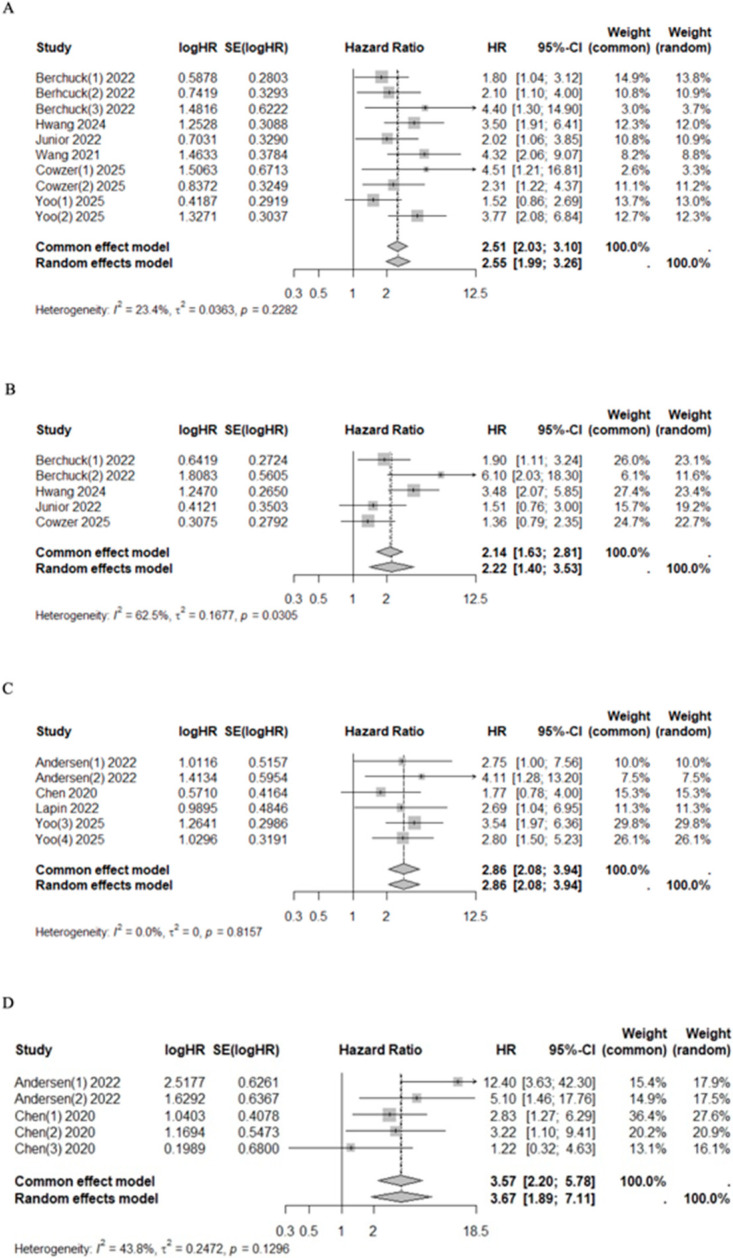
Forest plots of mortality [40,41,42,44,48,49] (**A**) and progression [41,44,48,49] (**B**) risk among patients who underwent pre-treatment and post-treatment ((**C**) for mortality [42,43,46,47] and (**D**) for progression [46,47]) sampling of ctDNA/cfDNA.

The pooled univariate HR for mortality and progression risk in cfDNA/ctDNA-positive patients collected at admission or pre-treatment compared with cfDNA/ctDNA-negative patients was 2.51 (95% CI 2.03–3.10, I^2^ = 23.4%, *p* = 0.2282) and 2.22 (95%CI 1.40–3.53, I^2^ = 62.5%, *p* = 0.0305), respectively (Figure 3). Similarly, after treatment, patients with positive ctDNA had higher mortality and progression risk with a pooled HR of 2.86 (95% CI 2.08–3.94, I^2^ = 0.0%, *p* = 0.8157) and 3.57 (95%CI 2.20–5.78, I^2^ = 43.8%, *p* = 0.1296), respectively (Figure 3). Significant heterogeneity was observed in the pre-treatment group for progression risk (I^2^ = 62.5%). However, the sensitivity analysis confirmed the prognostic value of cfDNA/ctDNA status, which remained consistent regardless of specific studies being included or excluded (Supplementary Figure S2).

### 3.4. Subgroup Analysis of Variant Allele Frequency

Five studies used VAF to define cfDNA/ctDNA positivity [41,43,44,48,49]. When reported, the median VAF ranged from 1.4% to 3.9%. When the median value was used as a cutoff, patients with VAF in ctDNA above the median value (VAF+) showed a decreased median OS (10.20 months, IQR 8.75–10.65 vs. 20.10 months, IQR 19.35–26.07; *p* = 0.002) and PFS (4.47 months, IQR 3.20–4.70 vs. 7.90 months, IQR 7.70–11.84, *p* = 0.01) compared with patients with a VAF below the median value (VAF−) (Table 5; Supplementary Figure S1).

**Figure 4:**
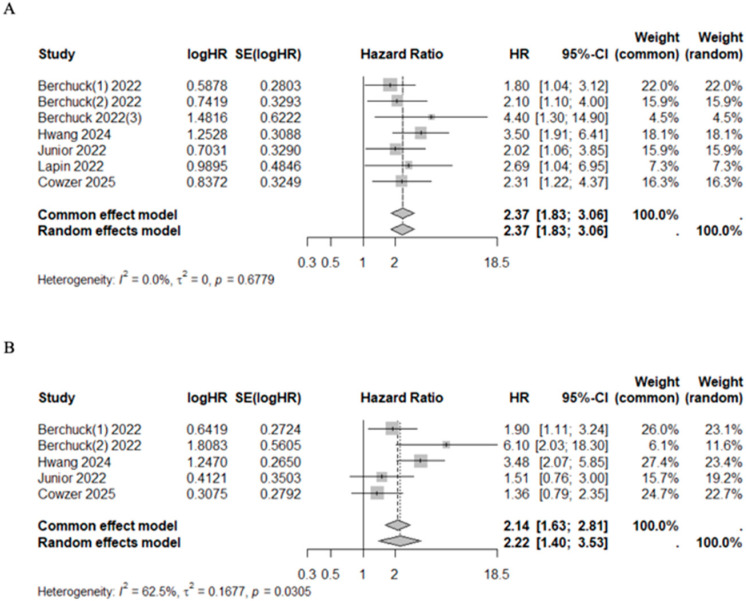
Forest plot on sub-group analysis on the VAF of ctDNA/cfDNA. (**A**) Overall survival [41,43,44,48,49]. (**B**) Progression free survival [41,44,48,49].

Patients with VAF+ had higher mortality and progression risk with a pooled HR of 2.37 (95%CI 1.83–3.06, I^2^ = 0.0%, *p* = 0.6779) and 2.22 (95%CI 1.40–3.53, I^2^ = 62.5%, *p* = 0.0305), respectively (Figure 4). Additionally, three studies classified patients into quartiles according to their maximum VAF value, reporting a median OS of 22.70 months (range 22.20–NR), 18.60 months (range 16.80–20.10), 14.10 months (range 9.30–18.30), and 9.00 months (range 7.00–11.80) for quartiles 1 through 4, respectively (Table 6) [44,48,49].

**Figure 5:**
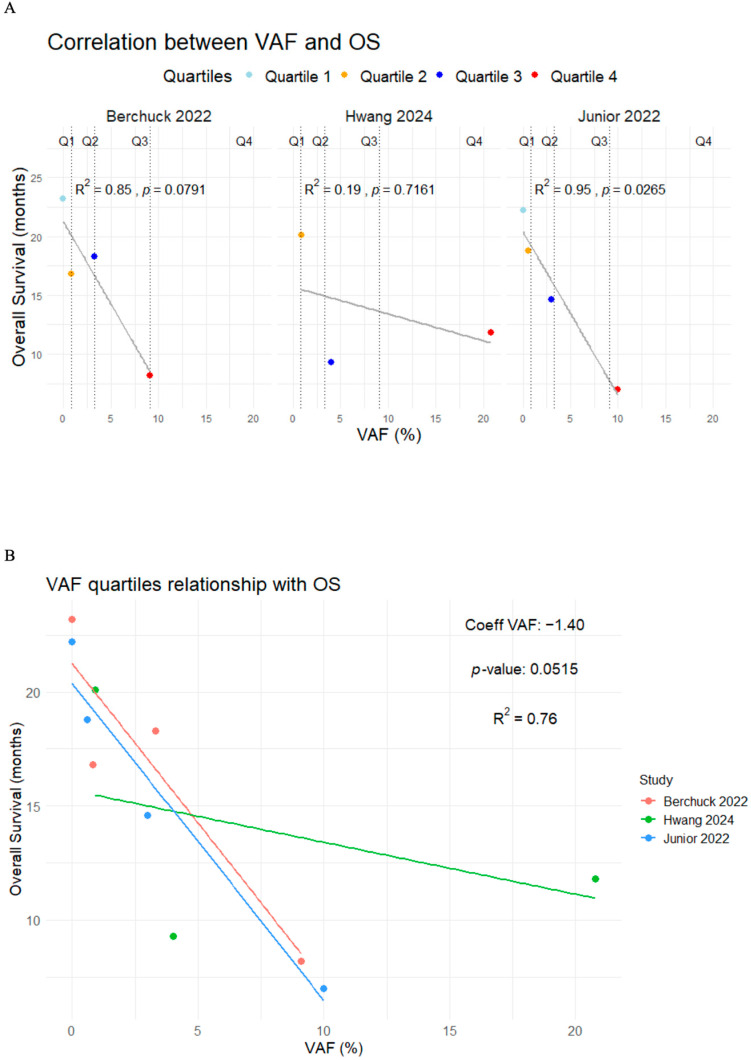
Correlation plots between the VAF quartiles and overall survival in the intra-group analysis (**A**) and between-group analysis (**B**) [44,48,49].

To better understand the potential relationship between the VAF and OS, intra-group and between-group correlation analyses across studies were conducted. In the intra-group analysis, Berchuck et al. [49] and Junior et al. [44] reported strong correlations (R^2^ = 0.85, *p* = 0.0791 and R^2^ = 0.95, *p* = 0.028, respectively), but only Junior et al. [44] provided statistically significant results. In contrast, Hwang et al. [48] reported a weak and non-significant correlation (R^2^ = 0.19; *p* = 0.7161). When analyzing the data across all studies, the relationship between VAF and OS remained strong, with an R^2^ value of 0.76 (*p* = 0.05) (Figure 5).

### 3.5. CfDNA/ctDNA to Assess Response to Treatment and Recurrence

**Figure 6:**
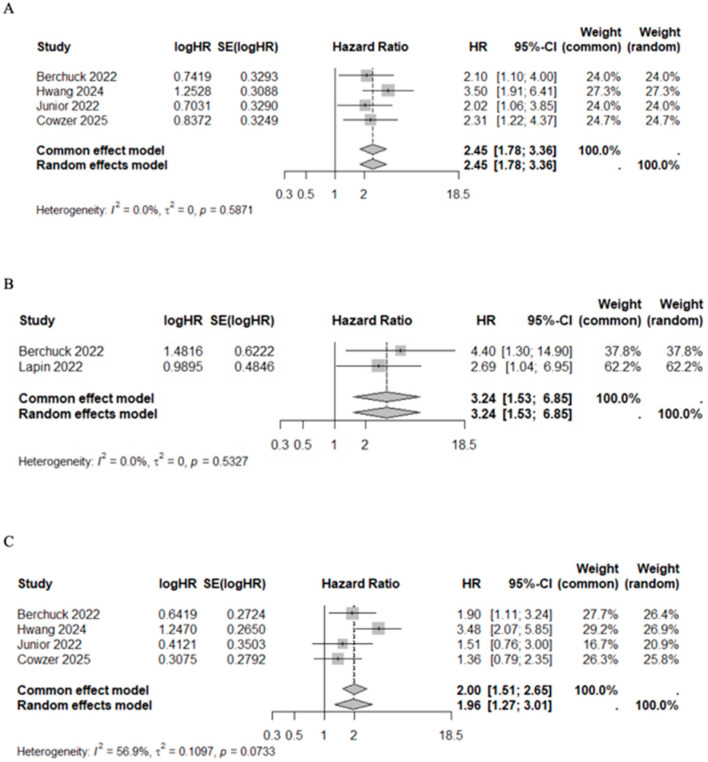
Forest plot of VAF+ versus VAF− patients under treatment for mortality risk after first line chemotherapy [41,44,48,49] (**A**) and targeted therapy [43,49] (**B**), and for progression risk after first line chemotherapy [41,44,48,49] (**C**).

In studies that evaluated the VAF below and above average, the pooled HR for mortality risk after first line chemotherapy (HR 2.45, 95%CI 1.78–3.36, I^2^ = 0%, *p* = 0.5871) and after target therapy (HR 3.24, 95%CI 1.53–6.85, I^2^ = 0%, *p* = 0.5327) were significantly higher among VAF+ patients. The association between VAF and progression risk prediction was addressed by four studies in patients receiving chemotherapy [41,44,48,49]. VAF+ patients had a higher progression risk compared with VAF− patients (HR 1.96, 95%CI 1.27–3.01, I^2^ = 56.9%, *p* = 0.0733) (Figure 6). Although significant heterogeneity was observed in the prediction of progression risk after chemotherapy (I^2^ = 56.9%), the sensitivity analysis confirmed the results regardless of whether the specific studies were included or excluded (Supplementary Figure S2). Median OS and PFS of patients with high VAF values under chemotherapy were 10.50 months (95%CI 9.57–11.61) and 4.58 months (95%CI 4.00–4.75), respectively. Conversely, among patients with low VAF values, median OS and PFS were 20.00 months (95%CI 19.62–23.09) and 9.77 months (95%CI 7.68–12.98), respectively (Table 7). This pattern was statistically significant for both OS (Mann–Whitney U test, *p* = 0.03) and PFS (Mann–Whitney U test, *p* = 0.03) (Supplementary Figure S1).

### 3.6. Recurrence and ctDNA/cfDNA

Two studies further investigated the relationship between ctDNA positivity and tumor recurrence; however, their findings were discordant [38,39]. Kim et al. [38] reported no significant correlation between the presence of SNV alterations in postoperative ctDNA and tumor recurrence (OR 0.67, 95%CI 0.11–3.92, *p* = 0.65) with a sensitivity and specificity of 44.44% and 45.45%, respectively. Conversely, Yu et al. [39] reported an association between ctDNA positivity and tumor recurrence with an OR of 162.00 (95%CI 13.36–1963.64) (*p* = 0.0001) and specificity and sensitivity of 93.8% and 94.9%, respectively (Supplementary Table S2).

## 4. Discussion

Biliary tract cancer (BTC) may benefit from early diagnosis as it is often asymptomatic in its initial stages and typically detected at an advanced stage, which is associated with a poor prognosis. This is further compounded by the lack of effective diagnostic tools, aggressive tumor biology, and the limited response to systemic therapy [30,50]. Additionally, tissue biopsy has several limitations, particularly in early stage disease, due to its invasiveness, the associated risk of complications, the need for specialized resources, and the potential for misdiagnosis or non-diagnostic results. Therefore, the trend toward personalized non-invasive medicine is increasing, with several studies investigating ctDNA in BTC, particularly in iCCA, which harbors actionable targets such as FGFR2 and IDH1 [29,51]. In their study, Winter et al. [52] reported the results of ctDNA measurements at various time points in four patients undergoing selective internal radiation therapy (SIRT) for metastatic iCCA. As a result of treatment response, a decrease in the burden of copy number variants (CNVs) was observed across serial ctDNA measurements. Several studies monitoring treatment response through ctDNA in breast and colorectal cancer and colorectal liver metastases reported similar results [16,53,54].

The clinical significance of ctDNA lies in its potential to serve as a diagnostic predictor, particularly in cases where conventional diagnostic approaches are inconclusive. Radiological assessments alone may be insufficient to provide a clear diagnosis of BTC, necessitating mandatory histological analyses [55,56] alongside laboratory markers such as CA19-9, which, on the other hand, can lack the specificity required for definitive diagnosis. Notably, up to 40% of BTC cases identified via endoscopic evaluation proceed to surgery without a conclusive diagnosis, and in 10% of these cases, no malignancy is detected in the surgical specimen [56,57]. In contrast, the PREVAIL ctDNA pilot trial demonstrated that ctDNA analysis can confirm BTC diagnosis in patients with suspected radiological findings with 75% specificity and 100% sensitivity [58]. Additional studies have reported a high specificity of ctDNA and related biomarkers in diagnosing and differentiating BTC [40,59,60]. Furthermore, several studies have highlighted ctDNA’s ability to reflect the tumor’s genetic landscape, reinforcing its role as a non-invasive surrogate for tumor tissue DNA, an essential feature supporting its clinical utility as a non-invasive diagnostic tool [48,61,62]. However, in the literature, the clinical relationship between BTC and ctDNA and its applicability is unclear.

This review demonstrated a direct correlation between elevated cfDNA/ctDNA levels and poorer long-term outcomes in patients with BTC, with a particular focus on cfDNA/ctDNA-associated biomarkers. Positivity of the ctDNA status was consistently predictive of a poor prognosis, regardless of the time of sampling. These findings align with earlier meta-analyses that examined the use of ctDNA for HCC. Specifically, Liu et al. [63] found ctDNA as a powerful predictor of survival in a cohort of 577 HCC patients. The present review collected studies in which the cfDNA/ctDNA of more than 2000 patients with BTC was collected before and/or after treatment as well as in treatment-naïve patients. Combined data for OS and PFS showed an association with worse outcomes, regardless of the sampling time point or treatment administration. Notably, subgroup analyses revealed that the timing of sampling relative to treatment may predict a poorer prognosis and be relevant for disease monitoring.

Additionally, VAF within ctDNA has also been identified as a potential marker for predicting clinical outcomes. Specifically, VAF is the proportion of sequencing reads in a ctDNA sample that support a specific genetic variant. In the context of BTCs, understanding VAF characteristics is crucial for identifying driver mutations and targeting treatments. High VAF values indicate that a significant proportion of cancer cells harbor a mutation, which may indicate a clonal driver mutation crucial to tumor growth. In line with the literature, the present review found that lower VAF values were associated with better overall and progression-free survival outcomes. In this regard, although Ettrich et al. [45] reported a non-statistically significant correlation in the overall CCA cohort (*p* = 0.0288, r = −0.5878; Spearman test), it became significant when analyzed at the subgroup level for ICC and ECC. Importantly, residual disease may be assessed by measuring VAF levels in ctDNA after surgery or systemic therapy. In this review, subgroup analysis showed that patients with VAF values above a certain threshold (the cohort median value) had an increased risk of shorter OS and PFS. While a stable or rising VAF value may suggest a higher risk of progression, declining VAF levels after treatment indicate that the therapy was effective. Consequently, we conducted a subgroup analysis stratified by treatment modality. This analysis revealed a significant association between ctDNA and unfavorable OS among patients receiving first line chemotherapy (HR 2.45, 95%CI 1.78–3.36, I^2^ = 0%, *p* = 0.5871) as well as those receiving targeted therapy (HR 3.24, 95%CI 1.53–6.85, I^2^ = 0%, *p* = 0.5327). VAF was reported as the only cfDNA feature independently associated with worse OS in one of the largest studies involving a well-characterized cohort of patients with solid tumors, whose medical data were thoroughly reviewed and linked to specific clinical information for each individual [64]. More precisely, the top quartile of maximum VAF (>8.6%) was considered a predictor of dismal prognosis [64], which is consistent with the quartiles analysis reported in the present review. Additionally, an important finding that emerged from this review is the potential utility of longitudinal ctDNA monitoring as a biomarker of treatment response. Whereas most studies to date have focused primarily on baseline ctDNA levels, dynamic changes in ctDNA during therapy may provide earlier and more accurate indicators of therapeutic efficacy. Yoo et al. [42] presented compelling proof-of-concept evidence demonstrating that patients who converted from ctDNA-positive to ctDNA-negative status during adjuvant chemotherapy experienced significantly improved DFS, comparable to that observed in patients who were ctDNA-negative at the baseline. Despite these encouraging observations, only a limited number of studies have reported longitudinal ctDNA or VAF measurements, and the considerable methodological heterogeneity among them currently precludes robust pooled analyses. Standardized, prospective investigations are therefore warranted to validate ctDNA dynamics as a predictive biomarker and to define clinically actionable thresholds. Such efforts could complement existing biomarkers and ultimately facilitate more individualized treatment strategies.

The role of ctDNA as an independent predictor of recurrence after curative-intent surgery has been reported for colorectal cancer [65], gastric cancer [66], and breast cancer [67]. Currently, postoperative surveillance for BTC relies primarily on radiological imaging, which may delay the detection of recurrence [68]. Emerging evidence from this review suggests that ctDNA holds significant promise as a biomarker for detecting recurrence in resected BTC. Lamarca et al. [69] reported a potential association between ctDNA positivity and the risk of recurrence in a cohort of patients with pancreatic–biliary malignancies. Although statistical significance was not reached, the study reported recurrence rates of 37.5% in ctDNA-negative patients versus 66.7% in ctDNA-positive patients. In the present review, the ability of ctDNA positivity to predict the recurrence risk was evaluated, although only two studies reported these data [38,39]. The results were highly inconsistent and contradictory, possibly due to the complexity of using ctDNA as a biomarker for recurrence detection, variations in ctDNA testing methods, patient demographics, study designs, and definitions of recurrence as well as the challenge of defining the optimal timing for ctDNA collection. Specifically, Yu et al. [39] reported that ctDNA detected recurrence in 93.8% of patients, with an average lead time of 3.70 months, highlighting its potential as an earlier and more sensitive surveillance tool compared with standard imaging. However, further studies involving larger cohorts are needed to fully establish the clinical utility of ctDNA as a non-invasive method for monitoring BTC recurrence.

Among the total cohort included in this review (n = 2347), only 272 patients underwent surgical resection. Of these, 115 patients underwent surgery alone [38,39,40,41], while 143 patients received surgery followed by adjuvant chemotherapy [39,42]. In this specific setting, the role of ctDNA as a prognostic biomarker remains of high clinical relevance but still shows heterogeneity across studies. According to Wang et al. [40], ctDNA positivity following surgery was associated with worse OS [HR: 4.32 (95% CI 2.06–9.08) *p* = 0.033]. In contrast, Kim et al. [38] reported no significant correlation between postoperative ctDNA positivity and recurrence (OR 0.67, 95%CI 0.11–3.92, *p* = 0.65), showing a sensitivity of 44% and a specificity of 45% in detecting clinical recurrence. Notably, in the study by Yu et al. [39], the most comprehensive of the included studies focusing on this aspect, ctDNA positivity was associated with tumor recurrence (OR 162.00, 95%CI 13.36–1963.64, *p* = 0.0001), with poorer RFS both before and after adjuvant chemotherapy. These findings suggest that ctDNA detection after curative-intent surgery may help identify patients with minimal residual disease and a higher risk of early relapse, potentially guiding the need for intensified surveillance or additional therapeutic strategies. However, the existing literature is extremely limited, and these subjects merit further investigation through larger prospective studies to define the role of postoperative ctDNA status in predicting recurrence after curative-intent surgery for BTC, enabling postoperative risk stratification and informed decision-making for patient management.

It is essential to address several limitations when considering the results reported by this review. First, the absence of comprehensively shared and accepted reliable markers for ctDNA positivity led to variation in this parameter between studies. Second, the variability of detection methods and the analysis of cfDNA instead of ctDNA in three studies. Third, despite subgroup and sensitivity analyses, moderate heterogeneity persisted, particularly for the risk of progression in overall ctDNA status and pre-treatment sampling subgroup analysis. This likely reflects both biological and methodological variability. Biologically, differences in tumor burden, disease stage, and ctDNA shedding dynamics across biliary tract cancer subtypes (intrahepatic, extrahepatic, gallbladder) may influence ctDNA detectability and prognostic strength. Methodologically, the use of diverse ctDNA platforms (e.g., digital PCR, NGS), variable positivity thresholds, and heterogeneous treatment settings further contribute to inconsistency. These factors likely underline much of the observed heterogeneity and emphasize the need for standardized ctDNA assays and reporting in future studies.

## 5. Conclusions

In conclusion, this review is the first to comprehensively explore the role of liquid biopsy in predicting the long-term outcomes in patients with BTC. The evidence supports ctDNA positivity as a strong predictor of poor long-term prognosis, independent of sampling time and therapy administration. Moreover, VAF emerged as a promising prognostic marker, with elevated levels significantly associated with worse survival. Importantly, ctDNA analysis offers a non-invasive means to detect residual disease after surgery or systemic therapy and provides valuable molecular insights for identifying actionable targets. These findings highlight the potential of liquid biopsy to refine risk stratification, enable early relapse detection, and guide personalized treatment strategies in BTC, ultimately paving the way for more tailored and effective patient care. However, the widespread clinical implementation of ctDNA analysis is currently limited by the high costs associated with testing, underscoring the need for strategies to improve accessibility and cost-effectiveness, ultimately paving the way for more tailored and effective patient care.

## Figures and Tables

**Table 1:** Baseline characteristics of the selected studies.

Study ID	Country	Year	Patients	Pt. with LB	Liquid Biopsy	Age	Treatment
			*n*	*n*		Median (Range)	
Ettrich 2019 [45]	Germany	NR	24	23	ctDNA	66.7 ± 12.2 *	CT = 23
Chen 2020 [47]	China	2018–2019	37	30	ctDNA	64.0 (41.0–74.0)	CT = 37
Wang 2021 [40]	China	2019–2020	47 ^§^	47 ^§^	cfDNA	59.0 (50.0–63.0)	TN = 47
Berchuck 2022 [49]	USA	2015–2019	1671	1671	cfDNA	65.0 (56.0–72.0)	TN = 105 ** CT = 86 ** TT = 29 **
Lapin 2022 [43]	USA	2015–2017	31	31	ctDNA	60.0 (35.0–77.0)	TT = 31
Junior 2022 [44]	USA	2016–2020	67	67	ctDNA	67.0 (27.0–90.0)	CT = 67
Andersen 2022 [46]	Denmark	2012–2017	39	39	ctDNA	62.0 (25.0–80.0)	CT = 39
Kim 2023 [38]	Korea	2018–2020	20 ^§^	20 ^§^	ctDNA	66.8 (35.0–79.0)	TN = 6 AT = 14
Hwang 2024 [48]	Korea	2019–2022	139	102	ctDNA	66.0 (59.0–71.0)	CT = 137 CT + IT = 2
Yu 2025 [39]	USA	2019–2023	56 ^§^	56 ^§^	ctDNA	64.0 (57.0–71.0)	AT = 43 TN = 13
Cowzer 2025 [41]	USA	2014–2022	170 (50 ^§^)	60	cfDNA	64.7 (56.5–72.4)	CT = 120 TN = 50
Yoo 2025 [42]	Korea	2017–2020	101 ^§^	89 ^§^	ctDNA	67.0 (47.0–76.0)	AT = 101

* Mean value with SD. ** Cohort with available data on clinical outcomes. ^§^ Patients who underwent resection for BTC. Abbreviations: NR, not reported; LB, liquid biopsy; AT, adjuvant therapy; CT, chemotherapy; IT, immune therapy; TN, treatment naïve; TT, targeted therapy.

**Table 2:** Tumor diagnosis and ctDNA/cfDNA characteristics.

	Tumor				ctDNA/cfDNA			
Study ID	ICC	ECC	GBC	Other	Source	Collection	Isolation	Detection
Ettrich 2019 [45]	13	10	0	0	Plasma	Pre- and post-treatment	QIAamp Circulating Nucleic Acid Kit (Qiagen, Hilden, Germany)	NR
Chen 2020 [47]	30			0	NR	Post-treatment	NR	Sequencing (not specified)
Wang 2021 [40]	5 ^§^	16 ^§^	8 ^§^	0	Plasma	Treatment-naïve	QIAseq cfDNA Extraction Kit (Qiagen, Hilden, Germany)	Low coverage WGS (Illumina, San Diego, CA, USA)
Berchuck 2022 [49]	1526		145	0	Plasma	Pre-treatment	NR	Target NGS (Guardant 360, Redwood City, CA, USA)
Lapin 2022 [43]	31		0	0	Plasma	Pre- and post-treatment	QIAamp Circulating Nucleic Acid Kit (Qiagen, Valencia, CA, USA)	ddPCR and NGS (Guardant Health, Redwood City, CA, USA)
Junior 2022 [44]	54	7	6	0	Plasma	Pre-treatment	NR	Target NGS (Guardant 360, Redwood City, CA, USA)
Andersen 2022 [46]	23	6	1	9	Plasma	Post-treatment	QIAsymphony DSP (Qiagen, Hilden, Germany)	ddPCR (Thermo Fisher Scientific, Waltham, MA, USA)
Kim 2023 [38]	14 ^§^	4 ^§^	0	2	Plasma	Pre- and post-treatment (surgery)	QIAamp MinElute Midi Kits (Qiagen, Hilden, Germany)	Target NGS (Illumina, San Diego, CA, USA)
Hwang 2024 [48]	50	27	25	0	Plasma	Pre-treatment	Maxwell^®^ RSC34 cfDNA Plasma Kit (Promega, Madison, WI, USA)	Target NGS (Illumina, San Diego, CA, USA)
Yu 2025 [39]	24 ^§^	12 ^§^	8 ^§^	0	Plasma	Pre- and post-treatment	QIAamp Circulating Nucleic Acid Kit (Qiagen, Valencia, CA, USA)	PCR-based NGS
Cowzer 2025 [41]	121	29	20	0	Plasma	Pre- treatment	NR	NGS (Illumina, San Diego, CA, USA)
Yoo 2025 [42]	0	101 ^§^	0	0	Plasma	Pre- and post-treatment	NR	Target NGS (Illumina, San Diego, CA, USA)

^§^ Patients who underwent resection for BTC. Abbreviations: NR, not reported.

**Table 3:** Long-term outcomes of patients with positive ctDNA/cfDNA.

Study ID	No. Pt	Population	Marker ctDNA+	Endpoint	Univariate HR (95%CI)	Multivariate HR (95%CI)
Chen 2020 [47]	30	Overall	ctDNA presence	PFS	2.83 (1.27–6.28), *p* = 0.007	NA
	30			OS	1.77 (0.78–3.99), *p* = 0.16	NA
	17	Responders		PFS	3.22 (1.10–9.40), *p* = 0.02	NA
	13	Non responders		PFS	1.22 (0.32–4.60), *p* = 0.77	NA
Wang 2021 [40]	28 ^§^	Overall	CNV > 4 aberrations	OS	4.32 (2.06–9.08) *p* = 0.033	NA
Berchuck 2022 [49]	105	Treatment naïve	VAF ≥ 9%	OS	1.80 (1.0–3.0) *p* = 0.041	NA
	86	First line CT		PFS	1.90 (1.10–3.20) *p* = 0.035	NA
	86			OS	2.10 (1.10–4.00) *p* = 0.03	NA
	29	TT		PFS	6.10 (2.10–18.90) *p* = 0.0012	NA
	29			OS	4.40 (1.30–14.90) *p* = 0.019	NA
Junior 2022 [44]	66	Overall	DCAF ≥ 3%	OS	2.02 (1.06–3.85), *p* = 0.03	NA
	66			PFS	1.51 (0.76–3.00), *p* = 0.24	NA
	66		DCAF ≥ 10%	OS	3.47 (1.43–8.39), *p* = 0.002	13.07 (1.2–142.32), *p* = 0.035
	66			PFS	8.33 (2.24–31.03), *p* < 0.001	NA
Lapin 2022 [43]	31	Overall	VAF IDH increasing	OS	2.69 (1.04–6.95), *p* = 0.03	NA
Andersen 2022 [46]	33	First CT cycle	Met-HOXA9 above median	PFS	12.4 (3.63–42.25), *p* < 0.001	NA
	33			OS	2.75 (1.00–7.55), *p* = 0.04	NA
	33	Second CT cycle		PFS	5.1 (1.46–17.71), *p* = 0.0047	NA
	33			OS	4.11 (1.28–13.21), *p* = 0.0105	NA
Hwang 2024 [48]	101	Overall	VAF > 3.9%	OS	3.5 (1.94–6.51), *p* < 0.0001	3.49 (1.72–7.08), *p* = 0.0005
	101			PFS	3.48 (2.07–5.85), *p* < 0.0001	3.57 (1.93–6.60), *p* < 0.0001
Yu 2025 [39]	30 ^§^	Before AT	ctDNA detected	RFS	26.00 (2.6–265.0), *p* < 0.0001	NA
	42 ^§^	After AT		RFS	20.00 (2.6–153.0), *p* < 0.0001	NA
Cowzer 2025 [41]	50 ^§^	Treatment naive	ctDNA detected	OS	4.51 (1.21–16.81), *p* = 0.117	NA
	60	Before CT	VAF ≥ 1.4%	OS	2.31 (1.22–4.36), *p* = 0.007	NA
				PFS	1.36 (0.79–2.36), *p* = 0.26	NA
Yoo 2025 [42]	89 ^§^	Before AT (MRD window)	ctDNA detected	DFS	1.80 (1.06–3.07), *p* = 0.029	NA
				OS	1.52 (0.86–2.70), *p* = 0.148	NA
	89 ^§^	Any time post-surgery		DFS	3.81 (2.22–6.54), *p* < 0.001	NA
				OS	3.77 (2.08–6.84), *p* < 0.001	NA
	88 ^§^	12 weeks after AT		DFS	7.72 (4.09–14.56), *p* < 0.001	NA
				OS	3.54 (1.97–6.35), *p* < 0.001	NA
	77 ^§^	24 weeks after AT		DFS	5.24 (2.75–9.97), *p* < 0.001	NA
				OS	2.80 (1.50–5.24), *p* = 0.001	NA

^§^ Patients who underwent resection for BTC. Abbreviations: NA, not available; CT, chemotherapy; VAF, variant allele frequency; DCAF, dominant clone allele frequency; CNV, copy number variation; MRD, measurable residual disease; OS, overall survival; PFS, progression-free survival; RFS, recurrence-free survival.

**Table 4:** Long-term outcomes relative to ctDNA/cfDNA status.

Study	Population	OS (Median, mo.)		1-Year OS (%)		PFS (Median, mo.)		1-Year PFS (%)	
		ctDNA+	ctDNA−	ctDNA+	ctDNA−	ctDNA+	ctDNA−	ctDNA+	ctDNA−
Chen 2020 [47]	Overall	9.10	13.00	53.16	29.50	4.30	7.30	6.10	23.60
	Responders	/	/	/	/	5.00	8.40	14.50	29.70
	Non responders	/	/	/	/	4.10	4.20	NR	NR
Wang 2021 [40]	Overall ^§^	7.79	NR	50.00	89.00	/	/	/	/
Andersen 2022 [46]	Overall	4.50	5.65	NR	5.85	1.85	2.72	NR	NR
	First cycle	2.49	5.20	NR	3.40	0.64	2.46	NR	NR
Berchuck 2022 [49]	Naïve	8.20	20.10	32.10	65.40	/	/	/	/
	Chemotherapy	7.70	19.90	28.35	60.71	2.60	7.60	18.46	25.67
	Targeted therapy	10.50	15.30	25.88	70.97	3.20	7.90	NR	20.13
Junior 2022 [44]	Overall	10.80	18.80	38.70	73.80	4.60	7.70	23.27	38.71
Lapin 2022 [43]	Overall	9.30	33.10	42.90	80.50	/	/	/	/
Hwang 2024 [48]	Overall	10.20	20.10	33.60	87.83	4.90	16.40	14.90	61.10
Cowzer 2025 [41]	Naïve	38.70	39.00	60.00	100.00	/	/	/	/
	Advanced disease before CT	14.05	32.04	51.00	78.50	4.47	11.84	15.90	43.00
Yoo 2025 [42]	Before AT (MRD window)	32.10	38.60	76.20	97.00	/	/	/	/
	Any time post-surgery	27.40	NR	86.20	95.00	/	/	/	/
	12 weeks after AT	19.20	48.50	73.50	98.50	/	/	/	/
	24 weeks after AT	29.32	49.70	89.20	96.60	/	/	/	/

^§^: Patients who underwent resection for BTC. Abbreviations: NR, not reached; OS, overall survival; PFS, progression-free survival.

**Table 5:** Correlation of VAFs and their threshold with OS and PFS.

Study ID	Population	Median VAF %	VAF	OS	PFS	VAF	OS	PFS
		IQR	%	Median mo., IQR		%	Median mo., IQR	
Junior 2022 [44]	Overall	3.0 (0–97)	>3.0	10.80 (15.00–18.90)	4.70	<3.0	18.80 (15.00-NA)	7.70
Berchuck 2022 [49]	TN	No data	>9.0	8.20	/	<9.0	20.10	/
	CT	No data	>9.0	7.70	2.60	<9.0	19.90	7.60
	TT	No data	>9.0	10.50	3.20	<9.0	15.30	7.90
Lapin 2022 [43]	Overall	2.2 (0–12.9)	Increasing VAF	9.30 (2.10–16.40)	/	Decreasing VAF	33.10 (13.00–53.20)	/
Hwang 2024 [48]	Overall	3.9	>3.9	10.20	4.90	<3.9	20.10	16.40
Cowzer 2025 [41]	Advanced disease before CT	1.2 (0.1–3.8)	≥1.4	14.05 (5.39–19.97)	4.47 (2.17–6.48)	<1.4	32.04 (13.82–39.93)	11.84 (5.39–18.42)

Abbreviations: VAF, variant allele frequency; CT, chemotherapy; TN, treatment naïve; TT, targeted therapy.

**Table 6:** Relationship between the variant allele frequency quartiles and overall survival.

Study ID	Quartile 1		Quartile 2		Quartile 3		Quartile 4	
	VAF Range (%)	OS (Median, mo.)	VAF Range (%)	OS (Median, mo.)	VAF Range (%)	OS (mo.)	VAF Range (%)	OS (Median, mo.)
Berchuck 2022 [49]	0.0 -0.7	23.20	0.8–3.2	16.80	3.3–9.0	18.30	9.1–92.0	8.20
Junior 2022 [44]	0.0–0.6	22.20	0.6–3.0	18.80	3.0–10.0	14.60	>10.0	7.00
Hwang 2024 [48]	0.0–0.8	NR	0.9–3.9	20.10	4.0–20.7	9.30	20.8–87.1	11.80

Abbreviations: VAF, variant allele frequency; OS, overall survival; NR, not reached.

**Table 7:** Long-term outcomes relative to VAF status in ctDNA/cfDNA.

Study ID	Therapy	OS (Months)		HR (95%CI)	PFS (Months)		HR (95%CI)
		VAF+	VAF−		VAF+	VAF−	
Junior 2022 [44]	CT	10.80	18.80	2.02 (1.06–3.85)	4.70	7.70	1.51 (0.24–3.00)
Berchuck 2022 [49]	CT	7.70	19.90	2.10 (1.10–4.00)	2.60	7.60	1.90 (1.10–3.20)
	TT	10.50	15.30	4.40 (1.30–14.90)	3.20	7.90	6.10 (2.10–18.90)
Lapin 2022 [43]	TT	9.30	33.10	2.69 (1.04–6.95)	/		/
Hwang 2024 [48]	CT	10.20	20.10	3.50 (1.94–6.51)	4.90	16.40	3.48 (2.07–5.85)
Cowzer 2025 [41]	CT	14.05	32.04	2.31 (1.22–4.36)	4.47	11.84	1.36 (0.79–2.36)

Abbreviations: VAF, variant allele frequency; OS, overall survival; CT, chemotherapy; TT, targeted therapy.

## Data Availability

No new data were created or analyzed in this study. Data sharing is not applicable to this article.

## Supplementary Materials

The following supporting information can be downloaded at: https://www.mdpi.com/article/10.3390/cancers17213451/s1, Supplementary Figure S1: Median OS and PFS in the ctDNA+ versus ctDNA− patients (A), VAF+ versus VAF− patients (B), VAF+ and VAF– chemotherapy patients (C); Supplementary Figure S2: Sensitivity analysis for progression risk for ctDNA positivity (A), for patients who underwent pre-treatment cfDNA/ctDNA sampling (B), and VAF+ patients after chemotherapy (C); Supplementary Table S1: Newcastle Ottawa Scale for Quality Assessment; Supplementary Table S2: Diagnostic value of ctDNA on prediction of recurrence.
