# Supplementary material for: Prognostic Role of Circulating DNA in Biliary Tract Cancers: A Systematic Review and Meta-Analysis
Source: Cancers (Basel). 2025 Oct 28;17(21):3451. doi: 10.3390/cancers17213451 (PMC12609761; doi:10.3390/cancers17213451)

Supplementary Material

Prognostic Role of Circulating DNA in Biliary Tract Cancers: A Systematic Review and Meta-Analysis

Sara Boggio, MS[1]; Laura Alaimo, MD[1]; Edoardo Poletto, MD[1]; Alberto Quinzii, MD[2]; Giada Scoccati, MD[1]; Mario De Bellis, MD, PhD[1]; Simone Conci, MD, PhD[1]; Tommaso Campagnaro, MD, PhD[1]; Andrea Ruzzenente, MD, PhD[1,*]

1 Department of Surgery, Dentistry, Gynecology, and Pediatrics, Division of General and Hepato-Biliary Surgery, University of Verona, University Hospital G.B. Rossi, Verona, Italy.

2 Department of Medicine, Division of Oncology, University of Verona, University Hospital G.B. Rossi, Verona, Italy.

* Correspondence: andrea.ruzzenente@univr.it; General and Hepato-Biliary Surgery; Department of Surgery, Dentistry, Gynecology, and Pediatrics; University of Verona; P.le L.A. Scuro 10, 37134 Verona, Italy; Tel. +39 045 8124411.

Supplementary Tables

Supplementary Table 1. Newcastle Ottawa Scale for Quality Assessment.

Study ID	Selection			Comparability		Outcome			Total
	Representativeness of exposed cohort	Sample size	Ascertainment of exposure	Control for important factors	Additional factors	Assessment of outcome	Correlation with outcome	Adequacy of survival data	
Ettrich 2019 [41]	1	1	1	1	1	1	0	0	6/8
Chen 2020 [43]	1	1	1	1	1	1	1	1	8/8
Wang 2021 [46]	1	1	1	1	0	1	1	1	7/8
Berchuck 2022 [47]	1	1	1	1	1	1	1	1	8/8
Lapin 2022 [39]	1	1	1	1	1	1	0	1	7/8
Junior 2022 [40]	1	1	1	1	1	1	0	1	7/8
Andersen 2022 [42]	1	1	1	1	1	1	1	1	8/8
Kim 2023 [38]	1	1	1	1	1	1	0	0	6/8
Hwang 2024 [44]	1	1	1	1	1	1	1	1	8/8
Yu 2025 [45]	1	1	1	1	1	1	1	1	8/8

Supplementary Table 2. Diagnostic value of ctDNA on prediction of recurrence.

Study ID	ctDNA	Relapse		Sn	Sp	PPV	NPV	OR 95%CI	RR 95%CI
		Yes	No						
Kim 2023 [38]	ctDNA+	4	6	44.44 % (4/9)	45.45 % (5/11)	40.0% (4/10)	50.0% (5/10)	0.67 (0.11-3.92)	0.80 (0.30-2.13)
	ctDNA-	5	5						
	p value							0.65	0.65
Yu 2025 [45]	ctDNA+	15	2	93.8% (15/16)	94.9% (37/39)	88.2% (15/17)	97.4% (37/38)	162.00 (13.36-1963.64)	12.43 (3.21-48.15)
	ctDNA-	1	37						
	p value							0.0001	0.0003

Abbreviations: Sn=sensitivity; Sp= specificity; PPV= positive predictive value; NPV= negative predictive value; OR=odds ratio; RR= relative risk.

Supplementary Figures

Supplementary Figure 1: Median OS and PFS in ctDNA+ versus ctDNA- patients (A), VAF+ versus VAF- patients (B), VAF+ and VAF- chemotherapy patients (C).

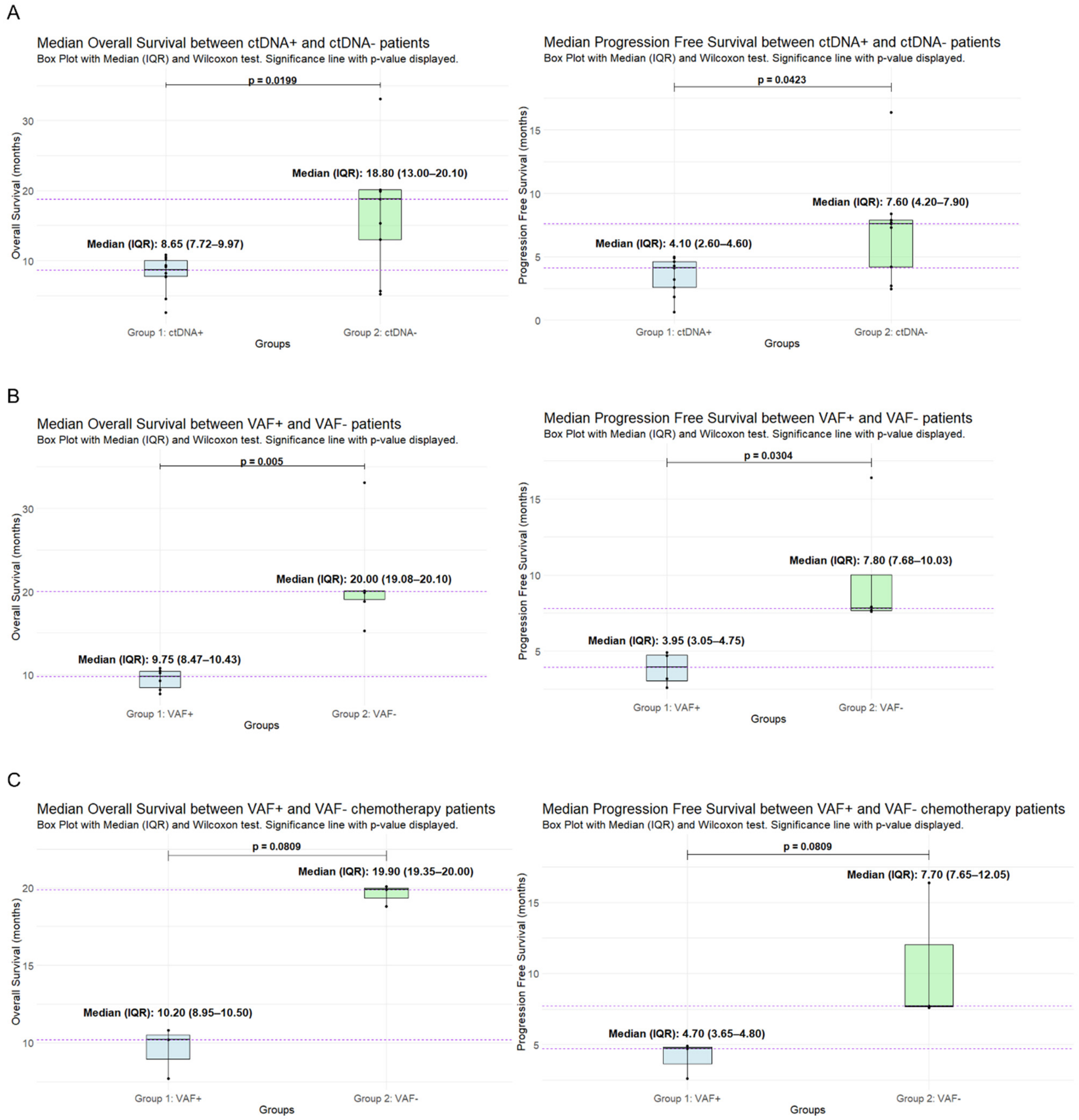

Supplementary Figure 2: Sensitivity analysis for progression risk of patients who underwent pre-treatment cfDNA/ctDNA sampling (A) and VAF+ patients after chemotherapy (B).

A

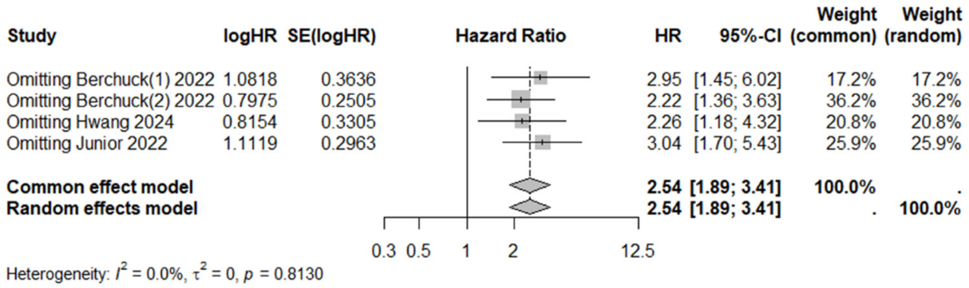

B

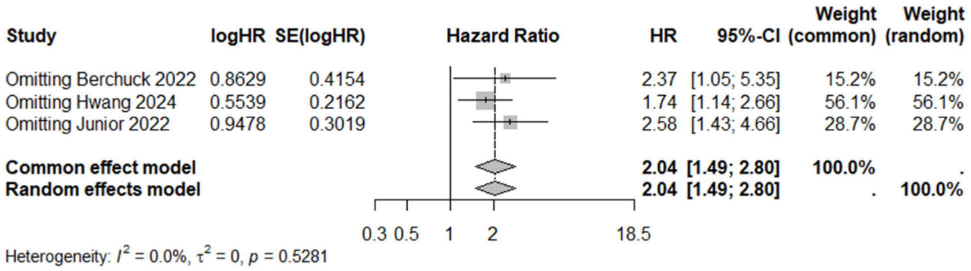

Supplement: Supplementary file 1 [file cancers-17-03451-s001.zip › cancers-3919127-supplementary.pdf]
